# Science of Psychometry

**Published:** 1889-03

**Authors:** 


### SCIENCE OF PSYCHOMETRY.

### PHENOMENAL PERCEPTIONS.

Sir John Lubbock, in his fascinating experiments with the myrmidons of the insect world, found that ants are highly sensitive to colors imperceptible to human vision. When a ray of light was dissected into a spectrum and cast upon his colony of tiny pets, the red extremity of the rainbow (to us the most effective) had no influence upon them; but as they were placed under the violet end they became much disturbed, and the dark portions beyond that limit of human observation tormented them into a frenzy of agitation. It appears that they do not appreciate light waves until they exceed the bounds of color—that is, of color visible to our eyes, though it is probable that the intensely active ultra-violet part of the spectrum, dark to us, is to them the most keenly brilliant of lights.

Further experiments in the effect of sounds upon these insects showed that they hear none of the noises that enter our ears. A pistol shot over them was unnoticed, except by the mechanical jarring of the air which it caused. These and similar tests have developed the conclusion that the insect world is wholly removed from the larger animals in its senses of color and sound, as of smell, taste and feeling, and that the human ideas of sense impressions are only a small section of the whole scheme of sense-life. It is understood by scientists that there are many strata of sight, hearing, etc., above and below the narrow plane of our own common perceptions. The insects are almost as far removed from us as spirits in the inconceivable fineness of their senses. They move among us in a wholly different world, seeing things that are concealed from us, hearing what is as silent as the stars to us, smelling in a way that to us is miraculous, feeling with an exquisite daintiness that to our gross experience is angelic. The birds and the denizens of the deep know many secrets of physical activity that exceed our ken. Even our own neighbors in the animal scale enjoy faculties that we cannot understand.

These instances serve to show the dulness of human senses in general and prepare us to appreciate the higher sensitiveness of some individuals. There is a form of superior acuteness in certain individuals which is seldom seen and therefore is commonly unknown ; but it is a curious indication of what higher development humanity is capable of even in its physical embodiment. It is all the more interesting because it is not, like Spiritualism, associated with trickery and collapse of character. It implies strength of soul rather than mediumistic weakness. Fortunately it is too rare to be made a profession.

The persons who are susceptible to this kind of impressions are set in nature's finest cases of flesh. Like a barometer they feel all the atmospheric changes, without any aid from the ordinary human weather indicators found in corns and gout. They are depressed and exhilarated in astonishing degrees by subtle movements, not only of the air, but of life. Feminine intuition is exalted in them to an infallible discernment of hidden things. Just as some people are peculiarly alert to the presence of certain odors or creatures, so these individuals seem to be a bundle of excessive sensibilities. But the experiences that these "sensitives" are most notable in are discoveries of scenes and impressions associated with the objects presented to them.

It is understood by students of science that the principles industriously applied in photography are vastly more far-reaching than the most magical solution of chemicals can trace. Physiologies record many examples of pictures impressed by special circumstances upon the retina of the eye forcibly enough to be hauntingly present before its vision, and even remaining on the tiny optic screen after death. A wide observation gathers the fact that the world is full of eyes, that everything is a camera seeing and recording all that comes before it, that only our ignorant blindness veils the myriad panoramas that attach to the objects around us,—panoramas that show in their order all the events that have taken place there.

The substantial basis of these apparently extravagant deductions is confirmed by the words of Dr. J. W. Draper, an unquestioned authority upon physic: "A sunbeam or a shadow cannot fall upon a surface, no matter of what material that surface is composed, without leaving upon it an indelible impression, and an impression which may, by subsequent application of proper chemical agents, be made visible. * * * Time seems to have so little influence on these effects that I conceive it possible, if a new vault should hereafter be opened in the midst of an Egyptian pyramid, for us to conjure up the swarthy forms of the Pharaonic officials who were its last visitors, though forty centuries may have elapsed since their departure."

The laboratory has not yet produced developing agents strong enough to bring forth such concealed pictures, but the phenomenal perceptions of "psychometers" accomplish more in this direction than materialistic science can do.

The word "Psychometry," whose meaning in this use (the measuring or tracings of things by the soul) does not appear in the dictionaries, comes from Dr. J. R. Buchanan, a Kentucky experimenter in these matters whose book, with this word as its title (published half a century ago) first presented in a scientific form many facts connected with the sensitiveness of some people to these hidden photographs. He hoped to establish a new science, by the aid of reliable assistants, which would draw the veil from many obscure departments of nature, and extend the bounds of knowledge in all directions. But the world is still unaware of his movements, though he has been observing and advocating and occasionally publishing ever since.

The best "psychometer" known to the writer is a modest little lady hidden away from the world in a great city. Only her friends know of her powers as she never exhibits to the public or exercises her gift for money. From childhood she has been abnormally sensitive to everything. Going into a room she feels at once the morale of its occupants. Touching a thing she receives strange impressions from it that are proven by investigation to be reminiscences of its history. Scenes are constantly coming before her, invisible to every one else, but to her as real as flesh and blood, generally attached to the persons or objects near her.

Her husband finds it difficult to conceal things from her, or to surprise her. She is not a spiritualist or a medium, and her whole life is fragrant with sweetest wholesomeness. Among circumstances that would be distressing to most persons she is always bright and cheerful. Her delicate physique smiles at pain and discomfort. Upon the circle of her friends she radiates her own buoyant and hopeful spirit, frequently reciting chapters of their past or glimpses of their future that elevates their courage above the commonplace grade of life. She stands like a kindly watchman high above the heads of the crowd, seeing forward and to the rear, far beyond the usual dim vision.

The bondage of physical existence rests lightly upon her, and the invisible verities open many shining doorways to her vision. She is an optimist because she sees the beneficent trend of all things. If a piece of writing is held in her hand, from a source unknown to her, she feels the influence of it strongly enough to describe the writer, and often the substance of the writer, in large, but accurate outline. There is nothing akin to mind reading, for she says things that puzzle the observer, though always true ; and there is no clairvoyance, for she cannot give the exact writing. Better illustrations of her peculiarities appear in the objects that are handed her to test. A scrap of mummy cloth, concealed from her sight in an envelope, brought forth a description of Egypt, a funeral service, the mountain tombs, a princely character and the Turks.

A piece of turquoise from Arizona produced scenery characteristic of that section, and Pueblo houses and ceremonies. Often she will be oppressed severely by the influence of some unpleasant spectacle or experience connected with the thing she is holding, and sometimes made to faint. Her method is simply to be passively receptive to what she is holding and watch the train of pictures arising from it.

A very curious and interesting book upon this subject was published twenty-five years ago, called "The Soul of things." It is the record of the investigation of the author, Prof. William Denton, some time connected with Harvard University, based upon many and long tests with several "psychometers" whose names are given, especially his own wife and son, who were marvellously sensitive in these perceptions.

The experiments were severely separated from elements likely to produce confused conclusions. The objects examined were always unexplained to the tester. A specimen will show his result.

A small black pearl, from the Gulf of California, was given to a lady to examine. She did not see it, but supposed it to be a small bean. She said: "I am traveling a long way. I see a man on his knees, stooping and scraping with his hands. He has something like a basket on a stick, which he puts into the water and dips out, and then scrapes with his hands in the dirt. It is not much inhabited here. He is getting stones or something valuable, for he is very choice of them, selecting them with great care. He scrapes into this basket full of holes; the water runs through, and he selects out of what remains at the bottom. They may be diamonds, for aught I know; they shine very bright. He puts them into a little bag. Back from him there is a large building, but it looks as if no one lived there. I see goats; they are the only animals that I can find. I see trees and vines."

Over a hundred experiments are recorded in this volume, all of these with geological, historic and archæological objects, whose known localities enabled a crucial test of accuracy, but in every case the object being utterly strange to the "psychometer." The results are surprisingly satisfactory. Often the same object was tried with several sensitives, and while the story is different for each the harmony and fitness are invariable.

The best result of psychometry is the realization that the world is a stupendous picture gallery. Nothing is lost or forgotten. All that has ever been lives in the memory of all that now is. It is impossible to escape the perpetuation of every act. The very atoms about us are watchful eyes that will tell the story to all coming ages. Not only is it true that every word goes ringing on eternally, like the increasing circles made by a pebble in a lake, but every minutest event is preserved indelibly. To a trained psychometer the long succession of histories seen by the thing he holds proceeds like the unbosoming of a long-traveled friend. And this little glance under the curtain of things eternal suggests how intimately related are all things, telling each other their hearts' secrets and all they have learned.—*The Cosmopolitan.*

